# The incomplete circle of Willis is associated with vulnerable intracranial plaque features and acute ischemic stroke

**DOI:** 10.1186/s12968-023-00931-2

**Published:** 2023-04-06

**Authors:** Huiying Wang, Lianfang Shen, Chenxi Zhao, Song Liu, Gemuer Wu, Huapeng Wang, Beini Wang, Jinxia Zhu, Jixiang Du, Zhongying Gong, Chao Chai, Shuang Xia

**Affiliations:** 1 The School of Medicine, Nankai University, Tianjin, 300071 China; 2 Department of Radiology, First Central Clinical College, Tianjin Medical University, Tianjin, 300192 China; 3 Department of Radiology, Tianjin Huanhu Hospital, Tianjin, 300350 China; 4 Department of Radiology, Affiliated Hospital of Inner Mongolia Medical University, Hohhot, 010000 China; 5 MR Collaboration, Siemens Healthineers Ltd., Beijing, 100102 China; 6 Department of Neurology, School of Medicine, Tianjin First Central Hospital, Nankai University, Tianjin, 300192 China; 7 Department of Radiology, School of Medicine, Tianjin First Central Hospital, Nankai University, Tianjin, 300192 China; 8 Tianjin Institute of Imaging Medicine, Tianjin, 300192 China

**Keywords:** Circle of Willis, Intracranial atherosclerosis, Magnetic resonance imaging, Ischemic stroke, Transient ischemic attack

## Abstract

**Background:**

The circle of Willis (CoW) plays a significant role in intracranial atherosclerosis (ICAS). This study investigated the relationship between different types of CoW, atherosclerosis plaque features, and acute ischemic stroke (AIS).

**Methods:**

We investigated 97 participants with AIS or transient ischemic attacks (TIA) underwent pre- and post-contrast 3T vessel wall cardiovascular magnetic resonance within 7 days of the onset of symptoms. The culprit plaque characteristics (including enhancement grade, enhancement ratio, high signal in T_1_, irregularity of plaque surface, and normalized wall index), and vessel remodeling (including arterial remodeling ratio and positive remodeling) for lesions were evaluated. The anatomic structures of the anterior and the posterior sections of the CoW (A-CoW and P-CoW) were also evaluated. The plaque features were compared among them. The plaque features were also compared between AIS and TIA patients. Finally, univariate and multivariate regression analysis was performed to evaluate the independent risk factors for AIS.

**Result:**

Patients with incomplete A-CoW showed a higher plaque enhancement ratio (*P* = 0.002), enhancement grade (*P* = 0.01), and normalized wall index (NWI) (*P* = 0.018) compared with the patients with complete A-CoW. A higher proportion of patients with incomplete symptomatic P-CoW demonstrated more culprit plaques with high T_1_ signals (HT_1_S) compared with those with complete P-CoW (*P* = 0.013). Incomplete A-CoW was associated with a higher enhancement grade of the culprit plaques [odds ratio (OR):3.84; 95% CI: 1.36–10.88, *P* = 0.011], after adjusting for clinical risk factors such as age, sex, smoking, hypertension, hyperlipemia, and diabetes mellitus. Incomplete symptomatic P-CoW was associated with a higher probability of HT_1_S (OR:3.88; 95% CI: 1.12–13.47, *P* = 0.033), after adjusting for clinical risk factors such as age, sex, smoking, hypertension, hyperlipemia, and diabetes mellitus. Furthermore, an irregularity of the plaque surface (OR: 6.24; 95% CI: 2.25–17.37, *P* < 0.001), and incomplete symptomatic P-CoW (OR: 8.03, 95% CI: 2.43–26.55, *P* = 0.001) were independently associated with AIS.

**Conclusions:**

This study demonstrated that incomplete A-CoW was associated with enhancement grade of the culprit plaque, and incomplete symptomatic side P-CoW was associated with the presence of HT_1_S of culprit plaque. Furthermore, an irregularity of plaque surface and incomplete symptomatic side P-CoW were associated with AIS.

**Supplementary Information:**

The online version contains supplementary material available at 10.1186/s12968-023-00931-2.

## Background

Intracranial atherosclerosis (ICAS) is the main cause of acute ischemic stroke (AIS) and transient ischemic attacks (TIA) [1, 2]. ICAS accounts for 30–50% of the AIS cases in Asian countries [3]. Neurologic disorders, including ischemic stroke represent major public health and economic burden worldwide [4, 5]. Furthermore, greater than 50% stenosis of the major intracranial arteries are associated with AIS or TIA [6, 7]. Computed tomography angiography, cardiovascular magnetic resonance (CMR) angiography (CMRA), and rotational digital subtraction angiography (DSA) studies have shown that the stenosis of intracranial vessels is a vital imaging-based marker in individuals suspected with TIA and stroke [8]. However, the complex pathological features of atherosclerotic plaques cannot be explained by the degree of lumen stenosis alone [9, 10]. Moreover, vessel remodeling contributes to the plaque burden in addition to stenosis. High-resolution vessel wall imaging (HR-VWI) is widely used to evaluate the morphology of the intracranial atherosclerotic plaques [8]. Several studies evaluated the degree of stenosis in the affected arteries using this imaging technique [11–16]. HR-VWI studies have improved the understanding regarding the relationship between vascular pathology and the plaque features of intracranial atherosclerosis.

The circle of Willis (CoW) is an anatomical structure that connects the anterior and posterior blood circulation in the brain and is incomplete in a majority of the individuals [17–19]. Stable cerebral blood flow is maintained under normal conditions despite significant differences in the CoW among individuals [20]. The relationship between the CoW variants and acute ischemia is unclear. De Caro et al. reported a higher prevalence of CoW variants in stroke patients [21]. Furthermore, recent studies have shown that geometric variations of the intracranial vessels affected the formation and progression of ICAS [22, 23]. It was reported that atherosclerosis plaques tended to occur in arterial branches, curvatures and bifurcations because these sites were characterized by low, disturbed or oscillating blood flow [24]. There are many arterial bifurcations and tortuous parts at the downstream (bilateral middle, anterior, and posterior cerebral arteries) of the CoW. Previous study demonstrated that incomplete CoW influenced distribution of the middle cerebral artery (MCA) plaques because of changes in the wall shear stress [25]. The variations in CoW play a decisive role in the development of atherosclerosis [20, 26]. However, the mechanisms by which variations in CoW cause downstream large vessel atherosclerosis have not been well characterized.

We hypothesized that the CoW variants were associated with different plaque characteristics and induced AIS in patients with symptomatic ICAS. Therefore, in the present study, we evaluated the association between the CoW variants and different culprit plaque features such as hyperintensity in T_1_-weighted image, irregularity, positive remodeling, and enhancement grade. We also assessed if the incomplete CoW and plaque features were independent predictors of AIS.

## Methods

### Patient selection

This retrospective study was approved by the Ethics Committee of Tianjin First Central Hospital which waived informed consent. A total of 441 patients were identified for this study. The inclusion criteria were as follows: (1) the patients were admitted through the emergency department at our institutiton between September 2016 and December 2021 due to clinical symptoms of AIS or TIA, and underwent head cardiovascular magnetic resonance (CMR) examinations within 7 days of admission. TIA was defined as a transient onset of neurological dysfunction caused by focal brain ischemia without new infarction on Diffusion-weighted image (DWI) [27]; (2) the patients were 18 years of age or older; (3) stenosis (30–99%) of the large intracranial arteries were confirmed by the time of flight CMR angiography (TOF-CMRA) or strategically acquired gradient echo CMRA (STAGE-CMRA) [27, 28]; (4) with no intravascular intervention before the high-resolution CMR vessel wall examination; (5) detection of atherosclerotic plaques by HR-VWI in at least one of the major intracranial arteries considered as the etiology of the ischemic events; (6) good imaging quality to evaluate plaques and stenosis; (7) the symptomatic side of TIA patients can be confirmed.

The exclusion criteria were as follows: (1) patients with a history of non-atherosclerotic cerebrovascular diseases such as arthritis, Moyamoya disease, aneurysm, or dissection; (2) co-existence of more than 30% stenosis in the internal carotid artery, basilar artery, vertebral artery, or common carotid artery uptream of the CoW; (3) evidence of cardioembolic and aortic arch atherosclerosis; (4) contraindications to CMR or gadolinium contrast agents. National Institute of Health Stroke Scale (NIHSS) scores of the patients were evaluated by a neurological physician (J.X.D. with 5 years of experience and blinded to the imaging information) on the day of the HR-VWI examination. To determine the symptomatic side, two neurological physicians (J.X.D. and Z.Y.G. with 5 and 20 years of experience, respectively) based on the neurodiagnostic tests and detailed histories to identify brain injury and its vascular genesis according to the statement of American Heart Association/American Stroke Association Stroke Council [27, 29]. They were both blinded to the imaging information. The major risk factors for ICAS, namely, hypertension, hyperlipidemia, diabetes mellitus, and history of smoking, were also recorded for all the study subjects.

### Imaging protocols

CMR was performed on a 3T scanner (MAGNETOM Prisma, Siemens Healthineers, Erlangen, Germany) equipped with the 64-channel head and neck coil. The patients were provided with head cushions and earplugs to stabilize their head and minimize motion artifacts. The patients underwent routine head CMR, CMRA, and STAGE CMRA for the intracranial arteries. Firstly, DWI acquisitions were performed to locate the AIS lesions. Then, CMRA and STAGE CMRA were used to confirm the stenosis sites in the intracranial vessels and determine the integrity of CoW as described in a previous study [30]. The stroke mechanisms were categorized and analyzed according to DWI and CMRA (presented in the Additional file 1). Sagittal pre-contrast HR-VWI was performed using the Inversion-recovery prepared sampling perfection with application-optimized contrast using different flip angle evolutions (IR-SPACE) to evaluate the characteristics of all intracranial plaques [31]. Dynamic susceptibility contrast-enhanced perfusion weighted imaging (DSC-PWI) was used to detection of perfusion abnormalities, especially for TIA patients [32]. It was performed by injecting the gadolinium-based contrast agent (Magnevist; Schering, Berlin, Germany) at a dose of 0.2 ml/kg at a rate of 4 mL/s. Then, post-contrast IR-SPACE was performed to evaluate the enhancement characteristics of all intracranial plaques. The imaging sequence parameters are listed in Additional file 1: Table S1.

### Image analysis

#### Plaque identification on HR-VWI

**Fig. 1:**
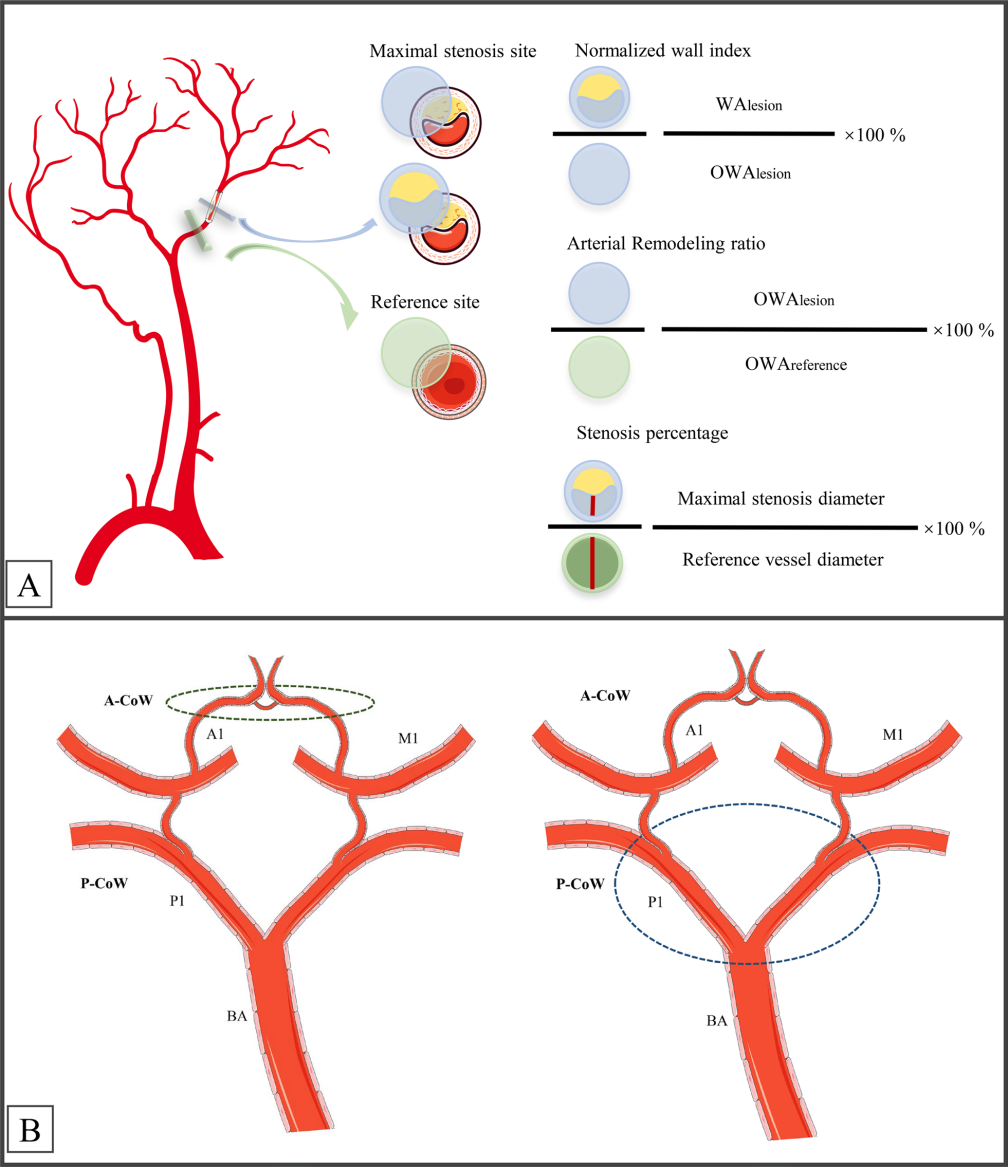
The schematic diagram of the circle of Willis (CoW) and the measurement formulas of lesions. **A** Methodology used to measure the normalized wall index, arterial remodeling ratio, and stenosis percentage. **B** Evaluation of the CoW. The CoW was divided into anterior- and posterior-CoW (A-CoW and P-CoW, respectively) sections. A-CoW consists of bilateral A1 segments and the anterior communicating artery. P-CoW consists of the bilateral P1 segments and the bilateral posterior communicating arteries. P-CoW was further categorized into incomplete and complete symptomatic sides

All the CMR images were analyzed independently by 2 neuroradiologists (H.Y.W. and C.X.Z. with 7- and 5-years’ experience, respectively), who were blinded to the clinical information of the patients. The symptomatic sides of TIA patients were confirmed through the neurodiagnostic tests, detailed clinical histories, and CMR imaging (vessel stenosis from CMRA, perfusion abnormalities from DSC-PWI) according to previous studies [27, 32] (a case presented in Additional file 1: Fig. S1). The HR-VWI source and multiplanar reformation (MPR) images displayed on the RadiAnt DICOM Viewer2021.1 workstation are shown in Additional file 1: Fig. S2. The observers carefully inspected the large intracranial arteries, including anterior cerebral arteries (up to the A2 segment), middle cerebral arteries (up to the M2 segment), and posterior cerebral arteries (up to the P2 segment) for the presence of plaques. When the thickness of the vessel wall was greater than 50% of the adjacent normal vessel wall in both the pre- and post-contrast IR-SPACE images [33, 34], the distinct area was defined as a plaque shown in Fig. 1. The reference vessel wall was defined as the adjacent proximal, distal, or contralateral cross-section with a thin, smooth vessel wall (Fig. 1**)**. The plaques were designated as culprit plaques based on the following criteria: (1) the only atherosclerotic lesion within the vascular territory of the stroke or TIA; or (2) the lesion with maximum stenosis when multiple plaques were present within the same responsible artery [15] (Additional file 1: Fig. S3). A plaque was considered as a non-culprit plaque when it was not within the vascular territory of the ischemic events. Plaques were categorized as culprit or non-culprit plaques by 2 independent neuroradiologists (G.M.W. and S.L. both with 7- years’ experience), who were provided with the clinical information, routine CMR images, and TOF-CMRA (or STAGE-CMRA) for the analysis. To avoid potential bias, they were not provided with HR-VW images. Quantitative and qualitative measurements of the individual plaques were performed using cross-sectional views of the HR-VWI images by two independent observers (H.Y.W. and C.X.Z. with 7- and 5-years’ experience, respectively). In case of any discrepancies between the two observers, the presence of a plaque was determined by a third senior observer (S.X. with 20-years’ experience in neuroradiology). After four weeks, one of the neuroradiologists (H.Y.W. with 7- years of experience) measured the quantitative and qualitative data for a cohort of 30 randomly chosen patients.

#### Evaluation of plaque burden and measurement of plaque features

The minimal lumen diameter at the site of maximal stenosis was measured for each stenosis vessel segment and divided by the diameter of the reference vessel wall to yield the stenosis percentage [15] (Fig. 1).The wall area of the lesion (WA_lesion_), the outer wall area of the lesion (OWA_lesion_), and the lumen area of the reference (LA_reference_) were measured using the formulas listed in Fig. 1. The normalized wall index (NWI) was calculated as WA_lesion_/ OWA_lesion_ ×100% [35]. The arterial remodeling ratio (ARR) was calculated as (OWA_lesion_/ OWA_reference_) ×100% (Fig. 1). ARR > 1.05 was considered as positive remodeling, whereas ARR < 0.95 was considered as negative remodeling [36].

**Fig. 2:**
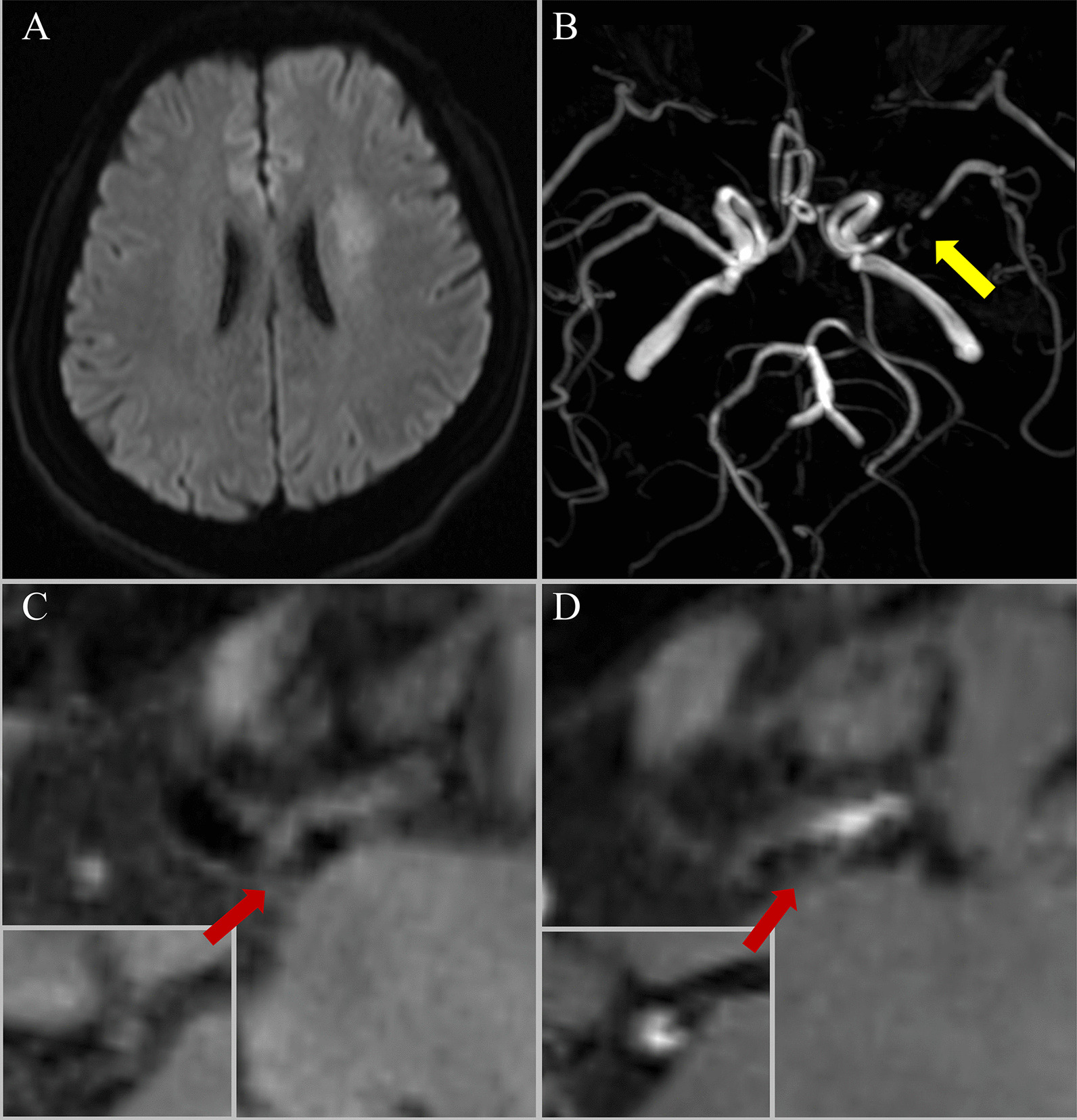
Representative case of a 58-year-old woman who was diagnosed with stroke before performing the CMR scan. **A** Diffusion weighted imaging (DWI) showed high signal intensity lesions on the left centrum ovale. **B** time of flight cardiovascular magnetic resonance angiography (TOF-CMRA) showed severe stenosis (yellow arrow) in the left middle cerebral artery (MCA) M1. **C**, **D** Curved multiplanar reconstructions of the **C** pre-contrast and **D** post-contrast HR-VWI showed a focal plaque in the left MCA with severe stenosis (stenosis ratio = 87.25%), irregular plaque surface, and grade 2 enhancement (plaque enhancement ratio = 3.13)

The mean signal intensity (SI) was measured within the regions-of-interest (ROIs), which were manually drawn in the brightest region of each identified plaque (SI_plaque_), the reference vessel wall (SI_ref_), and the pituitary infundibulum (SI_infund_) (Additional file 1: Fig. S4). The plaque signal in T_1_ weighted image was considered when the signal intensity was greater than 150% (SI > 150%) relative to the signal of the reference wall in the pre-contrast IR-SPACE [15]. The contrast enhancement ratio (CER) was quantified with the slice of greatest enhancement using the contralateral side of the brain parenchyma to normalize the signal intensity. The contrast enhancement ratio was estimated with the following formula: [(signal of plaque _post−contrast_/signal of contralateral brain parenchyma_post−contrast_)/ (signal of plaque_pre−contrast_/signal of contralateral brain parenchyma_pre−contrast_)] ×100% [37]. The lesion enhancement was classified into the following grades based on the post-contrast IR-SPACE: grade 0, SI_plaque_ ≤ SI_ref_; grade 1, SI_ref_ < SI_plaque_ <SI_infund_; grade 2, SI_plaque_ ≥ SI_infund_. We also recorded the plaque surface irregularity (defined as discontinuity of the plaque inner surface; Fig. 2) and regularity (smooth inner surface; Additional file 1: Fig. S5).

#### Analysis of the circle of Willis

The axial STAGE-CMRA source images containing 64 slices were obtained by post-processing the STAGE images using the STAGE software 1.2.1 (SpinTech, Bingham Farms, Michigan, USA) [28]. The structural characteristics of the CoW were evaluated using STAGE-CMRA or TOF-CMRA if either was available and STAGE-MRA if both were available. The type of CoW was analyzed by the two neuroradiologists (G.M.W. and S.L. with 7- years’ experience) using the original images as well as MIP images according to previously published criteria [30]. The anatomical structures of the CoW were divided into the anterior and posterior sections (Fig. 1) to determine the following types: (1) for the anterior CoW (A-CoW) section, type I with complete and normal anterior circulation vessels; type II with incomplete or dysplasia in the anterior half; (2) for the posterior CoW (P-CoW) section, type III with complete and normal in the symptomatic side of the posterior half (defined as normal and complete components of the P-CoW in the symptomatic side of the patients with clinical symptoms); type IV, incomplete or dysplasia in the symptomatic side of the posterior half (Fig. 1).

### Statistical analysis

The statistical data analysis was performed using the SPSS (version 22.0, Statistical Package for the Social Sciences, International Business Machines, Inc., Armonk, New York, USA) and GraphPad Prism (version 8.3.0, Graph-Pad Software, San Diego, California, USA) software. The quantitative data are presented as means ± standard deviation (SD) for normally distributed data or median (interquartile range, IQR) for data with skewed distributions. Categorical variables were expressed as counts (percentages). The intraclass correlation coefficient (ICC) (for continuous variables)/ Weighted kappa (for categorical variables)/ Cohen’s kappa (for binary variables) were conducted to measure inter-rater reliability and intra-reader reproducibility. The statistical differences in the culprit plaque features and the clinical data between patient groups with different types of CoW were determined using the two independent sample t-test for normally distributed quantitative data, the Mann-Whitney U-test for skewed distributions data, and the chi-square test or Fisher’s exact test for categorical data. Univariate binary logistic analysis was used to evaluate the association between different types of CoW and plaque characteristics such as the presence of high T_1_ signal (HT_1_S), irregularity of plaque surface, and positive remodeling. Univariate ordinal regression analysis was performed to determine the association between the types of CoW and culprit plaque enhancement grade. The variables with *P*-value < 0.1 were included in the multivariate regression analysis, which was conducted by adjusting for age, sex, and clinical risk factors, including a history of smoking, hyperlipemia, hypertension, and diabetes mellitus.

The differences in the culprit plaque characteristics between the AIS and TIA groups were compared using the two independent sample t-test for normally distributed quantitative data, Mann-Whitney U-test for skewed distributions data, and chi-square test or Fisher’s exact test for the categorical data. Univariate logistic analysis was performed to identify the variables associated with AIS, and those with *P*-value < 0.1 were included in multivariate analysis. Multivariate regression analysis was performed by adjusting for age, sex, and clinical risk factors, including a history of smoking, hyperlipemia, hypertension, and diabetes mellitus. Because patients with ischemic incidents may be identified with more than one plaque. The relationships between plaque characteristics and AIS were assessed using univariate and multivariate generalized estimating equations for patients with multiple intracranial plaques. A two-sided *P* < 0.05 was considered statistically significant. The receiver operating characteristic curves were performed to analyze the independent significance parameters.

## Results

### Demographics and basic characteristics of the study subjects

**Fig. 3:**
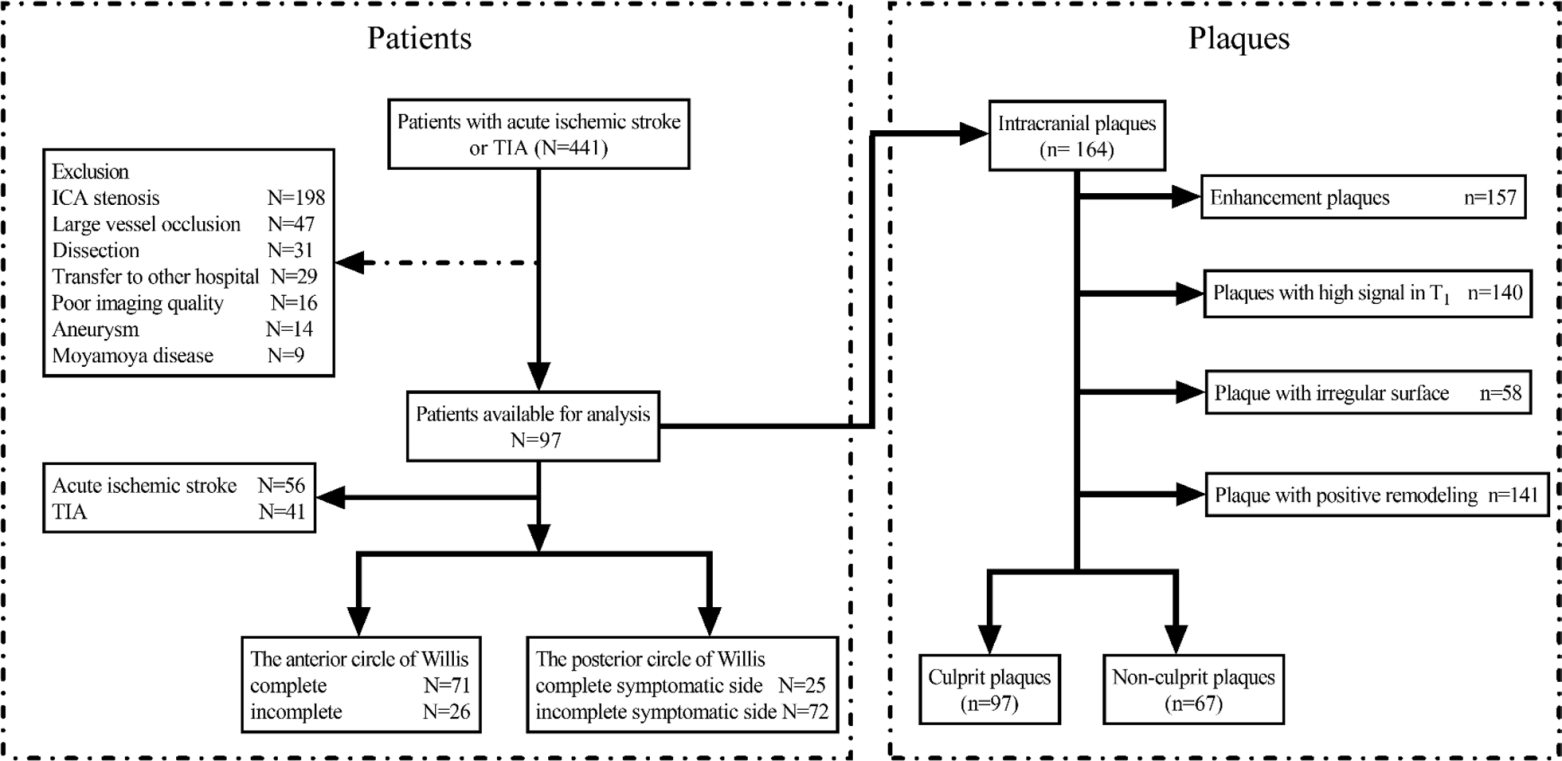
Flowchart of the study subjects and classification of the vessel lesions

This study included 97 patients, including (62 males, age 54.1 ± 14.0 years; age range: 23–80 years old). The A-CoW was incomplete in 26 (26.8%) of the 97 AIS or TIA patients, whereas the symptomatic side P-CoW was incomplete in 72 (74.2%) of 97 (Fig. 3). DWI hyperintensity was observed in 56 patients after symptoms onset, whereas the remaining 41 patients did not show acute lesions on the DWI. In the 97 patients, 164 intracranial plaques were identified, including 97 culprit plaques and 67 non-culprit plaques (Fig. 3). The degree of stenosis caused by the culprit plaque in our study ranged from 43 to 96%. The status of the clinical risk factors and the NIHSS scores of the study patients are shown in Table 1. We did not observe any significant differences in age, sex, and clinical risk factors between the AIS and TIA patients (all *P*>0.05). However, patients with AIS [2(1, 3.75)] showed higher NIHSS scores than the patients with TIA [0(0, 2)] (*P* < 0.001; Table 1). Furthermore, this study showed significantly high inter-rater and intra-reader reliability for the plaque characteristics and arterial remodeling ratio (0.73–0.99) (Additional file 1: Table S2).

**Table 1:** Demographics characteristics and culprit plaque feature between AIS and TIA patients

Characteristics	AIS (N = 56)	TIA (N = 41)	*P*
Age (years old)	53.1 ± 13.4	55.5 ± 14.9	0.418
Sex (Female/Male)	20/36	15/26	0.930
Clinical history [N (%)]			
Hypertension	39 (69.6%)	34 (82.9%)	0.134
Hyperlipidemia	26 (46.4%)	22 (53.7%)	0.482
Diabetes Mellitus	22 (39.3%)	17 (41.5%)	0.829
Ischemic heart disease	8 (14.3%)	5 (12.2%)	0.765
History of stroke	17 (30.4%)	18 (43.9%)	0.17
Smoking	28 (50%)	20 (48.8%)	0.906
NIHSS scores [median (IQR)]	2(1, 3.75)	0(0, 2)	<0.001*
CoW integrity [N (%)]			
Incomplete A-CoW	16 (28.6%)	10 (24.4%)	0.648
Incomplete S-P-CoW	49 (87.5%)	23 (56.1%)	**<0.001**^#^
Plaque features			
Stenosis percentage	69.3 ± 13.2	67.3 ± 14.0	0.476
Plaque enhancement ratio	202.6 ± 55.2	179.2 ± 59.7	**0.048**^^^
Enhancement grade (0/1/2)	0/36/20	4/30/7	**0.010**^#^
Irregularity of plaque [N (%)]	34 (62.5%)	10 (24.4%)	**<0.001**^#^
NWI	84.9 ± 11.1	83.9 ± 13.3	0.977
Positive remodeling [N (%)]	12 (21.4%)	4 (9.8%)	0.102
Arterial remodeling ratio	0.87 ± 0.29	0.79 ± 0.22	0.189
HT_1_S [N (%)]	51 (91.1%)	32 (78.0%)	0.073

*AIS *acute ischemic stroke, *TIA *transient ischemic attack, *A-CoW *anterior circle of Willis, *S-P-CoW * symptomatic side posterior circle of Willis, *NWI* normalized wall index, *HT*_*1*_*S* high T_1_signal

### Culprit plaque features and the type of CoW among AIS and TIA groups

The culprit plaques were more frequently observed in patients with AIS and showed higher enhancement grades (*P* = 0.01) and CER (*P* = 0.048) compared with the patients with TIA (Table 1). Furthermore, irregularity of the culprit plaque surface was more commonly detected in the patients with AIS compared with the patients with TIA (*P*<0.001) (Table 1). The presence of HT_1_S in the culprit plaques of AIS patients was slightly higher than that of TIA patients (*P* = 0.073). However, we did not observe any significant differences in the arterial remodeling ratio (*P* = 0.189), stenosis percentage (*P* = 0.476), NWI (*P* = 0.977), and positive remodeling (*P* = 0.102) between the two groups. A higher percentage of AIS patients showed incomplete symptomatic side P-CoW compared with the TIA patients (*P* < 0.001). (Table 1).

### Distinct characteristics of culprit plaque features in patients with incomplete anterior or posterior CoW

**Fig. 4:**
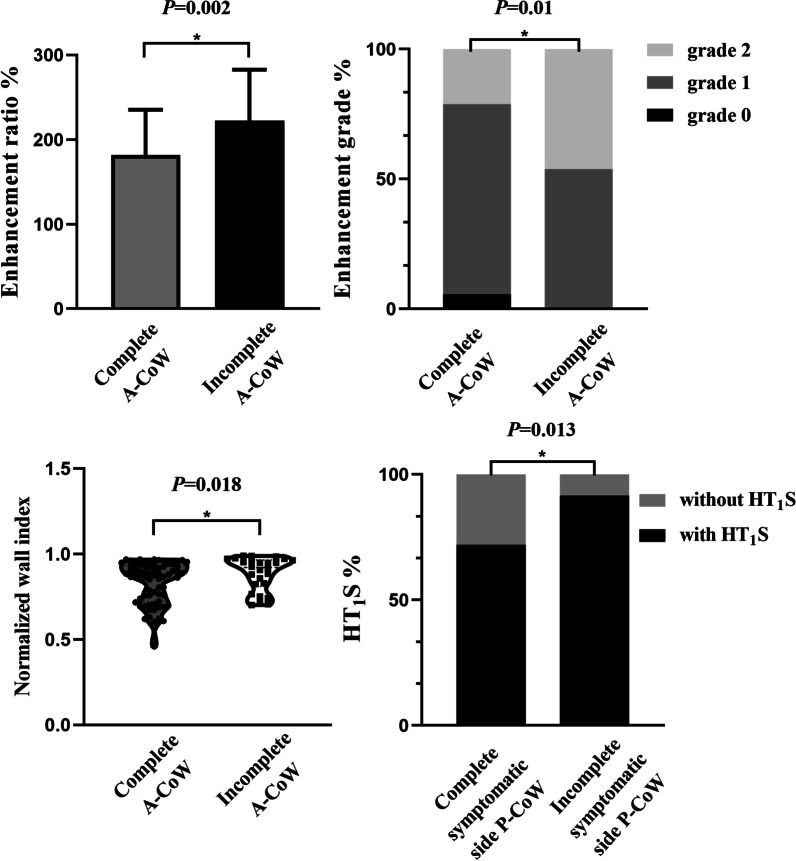
Comparison of the plaque characteristics between patients with different types of CoW. (Top) In the A-CoW group, patients with incomplete A-CoW showed a higher enhancement ratio (*P* = 0.002), and enhancement grade (*P* = 0.01) compared with the patients with complete A-CoW. (Bottom left) Patients with incomplete A-CoW also showed a higher normalized wall index (NWI) compared with those with complete A-CoW (*P* = 0.018). (Bottom right) In the P-CoW group, patients with incomplete symptomatic side P-CoW showed a higher probability of high T_1_ signal (HT_1_S) compared with those with complete symptomatic side P-CoW (*P* = 0.013)

The culprit plaques in the patients with incomplete A-CoW showed a higher CER (*P* = 0.002), enhancement grade (*P* = 0.01), and NWI (*P* = 0.018) compared with the patients with complete A-CoW (Table 2). However, culprit plaque features such as stenosis percentage (*P* = 0.538), HT_1_S (*P* = 0.730), irregularity of plaque (*P* = 0.925), arterial remodeling ratio (*P* = 0.890), and positive remodeling (*P* = 0.345) were statistically similar between the patients with complete and incomplete A-CoW (Table 2; Fig. 4). The culprit plaques in the patients with incomplete symptomatic side P-CoW showed a higher probability of HT_1_S compared with the patients with complete symptomatic side P-CoW (*P* = 0.013; Table 2). The culprit plaque features such as stenosis percentage (*P* = 0.247), CER (*P* = 0.610), enhancement grade (*P* = 0.931), irregularity of plaque surface (*P* = 0.760), NWI (*P* = 0.895), arterial remodeling ratio (*P* = 0.501), and positive remodeling (*P* = 0.987) did not show any significant differences between the patients with complete and incomplete P-CoW (Table 2; Fig. 4).

**Table 2:** Comparison of culprit plaque features between different types of CoW

Culprit plaque features	Complete A-CoW	Incomplete A-CoW	*P*	Complete symptomatic side P-CoW	Incomplete symptomatic side P-CoW	*P*
Stenosis percentage	67.9 ± 13.9	68.8 ± 12.4	0.538	71.1 ± 14.2	67.5 ± 13.2	0.247
Plaque CER	181.8 ± 53.6	222.5 ± 60.1	**0.002**^**#**^	193.7 ± 50.9	192.4 ± 60.6	0.610
Enhancement grade (0/1/2)	4/52/15	0/14/12	0.010*	0/19/6	4/47/21	0.931
HT_1_S [N (%)]	62(87.3%)	21(80.8%)	0.730	18(72.0%)	65(91.6%)	**0.013**^**&**^
Irregularity of plaque [N (%)]	32(45.1%)	12(46.12%)	0.925	12(48.0%)	32(44.5%)	0.760
NWI	83.0 ± 12.5	88.5 ± 9.8	**0.018**^**#**^	83.5 ± 14.3	84.8 ± 11.3	0.895
ARR	0.84 ± 0.26	0.82 ± 0.25	0.890	0.82 ± 0.29	0.84 ± 0.25	0.501
Positive remodeling [N (%)]	14(19.7%)	2(7.7%)	0.345	4(16.0%)	12(16.7%)	0.987

*A-CoW* anterior circle of Willis, *P-CoW *posterior circle of Willis, *ER *contrast enhancement ratio, *HT*_*1*_*S* high T_1_ signal; ^#^Independent samples t-test, *Fisher’s exact test，^&^chi-square test

### Incomplete CoW types associated with specific plaque features

Incomplete A-CoW was independently associated with enhancement grade of the culprit plaques before (OR:3.40; 95% CI: 1.32–8.76; *P* = 0.011) and after adjusting for clinical risk factors such as age, sex, smoking, hypertension, hyperlipemia, and diabetes mellitus (OR:3.84; 95% CI: 1.36–10.88; *P* = 0.011) (Table 3). Incomplete symptomatic side P-CoW was associated with the presence of culprit plaque HT_1_S before (OR:3.61; 95% CI: 1.12–11.61; *P* = 0.032) and after adjusting for clinical risk factors such as age, sex, smoking, hypertension, hyperlipemia, and diabetes mellitus (OR: 3.88; 95% CI: 1.12–13.47; *P* = 0.033) (Table 3).

**Table 3:** Association between incomplete CoW and culprit plaque features

Incomplete CoW	Univariate regression			Multivariate regression		
	OR	95% CI	*P*	OR	95% CI	*P*
HT_1_S						
A-CoW	1.64	0.49–5.45	0.419	1.555	0.40–6.00	0.522
Symptomatic side P-CoW	3.61	1.12–11.64	**0.032**	3.878	1.12–13.47	**0.033**
Enhancement grade						
A-CoW	3.40	1.32–8.76	**0.011**	3.840	1.36–10.88	**0.011**
Symptomatic side P-CoW	0.28	1.11–6.96	0.924	0.873	1.54–4.94	0.991
Positive remodeling						
A-CoW	0.29	0.04–2.17	0.226	0.134	0.01–1.34	0.087
Symptomatic side P-CoW	0.86	0.12–5.94	0.876	1.168	0.15–9.11	0.882
Irregularity of the plaque surface						
A-CoW	1.05	0.42–2.57	0.924	1.045	0.40–2.75	0.929
Symptomatic side P-CoW	1.15	0.46–2.87	0.758	1.215	0.48–3.09	0.682

*OR* odds ratio, *CI* confidence interval, *A–CoW* anterior circle of Willis, *P–CoW* posterior circle of Willis, *HT*_*1*_*S* high T_1_ signal

### Association between culprit plaque features, types of CoW, and AIS

The univariate logistic regression analysis showed that the culprit plaque features such as irregularity of plaque surface (OR: 4.79, 95% CI: 1.96–11.69, *P* = 0.001), enhancement ratio (OR: 1.01, 95%C I: 1.00–1.02, *P* = 0.052), enhancement grade 2 (OR: 2.38, 95% CI: 0.89–6.39, *P* = 0.085), and incomplete symptomatic side P-CoW (OR: 5.90, 95% CI: 2.07–16.87, *P* = 0.001) were associated with AIS. The irregularity of culprit plaque surface (OR: 6.24, 95% CI: 2.25–17.37, *P* < 0.001) and incomplete symptomatic side P-CoW (OR: 8.03, 95% CI: 2.43–26.55, *P* = 0.001) were independently associated with AIS after adjustment for age, sex, and clinical risk factors such as smoking, hypertension, hyperlipemia, and diabetes mellitus (Table 4). ROC curve analysis showed that the area under the ROC curve (AUC) value was 0.682 (95% CI, 0.574–0.790) for irregularity of the plaque surface, 0.654 (95% CI, 0.540–0.768) for incomplete P-CoW, and 0.774 (95% CI, 0.681–0.867) for a combination of these two factors with a sensitivity of 90.2% and a specificity of 53.6%.

**Table 4:** Association between vascular risk factors and acute ischemic stroke

Culprit plaque features	Univariate regression			Multivariate regression		
	OR	95% CI	*P*	OR	95% CI	*P*
Irregularity of plaques surface	**4.791**	**1.963–11.690**	**0.001**	**6.244**	**2.245–17.368**	**< 0.001**
Enhancement ratio (%)	**1.007**	**1.000-1.015**	**0.052**			
Enhancement grade 1	1.003	0.156–1.128	0.927			
Enhancement grade 2	**2.381**	**0.887–6.393**	**0.085**			
HT_1_S	0.349	0.107–1.134	0.129			
ARR (%)	0.292	0.056–1.516	0.143			
Incomplete S-P-CoW	**5.903**	**2.065–16.873**	**0.001**	**8.033**	**2.430–26.550**	**0.001**

*S-P-CoW* symptomatic side posterior circle of Willis, *HT*_*1*_*S* high T_1_signal, *ARR *arterial remodeling ratio, *OR *odds ratio, *CI *confidence interval

### Association between features of all plaques, types of CoW, and AIS

Irregularity of plaque surfaces (OR: 1.08, 95% CI: 1.03–1.13, *P* = 0.002), arterial remodeling ratio (OR: 1.06, 95% CI: 1.00-1.13, *P* = 0.043), enhancement ratio (OR: 1.00, 95% CI: 1.00–1.00, *P* = 0.070), enhancement grade 2 (OR: 1.23, 95% CI: 1.00-1.53, *P* = 0.064), positive remodeling (OR: 1.04, 95% CI: 1.01–1.07, *P* = 0.024), and incomplete symptomatic side P-CoW (OR: 5.92, 95% CI: 2.07–16.92, *P* = 0.001) were associated with AIS for all the intracranial plaques. Irregularity of plaque surfaces (OR: 1.01, 95% CI: 1.00–1.02, *P* < 0.001), enhancement grade 2 (OR: 1.03, 95% CI: 1.00–1.07, *P* = 0.048), and incomplete symptomatic side P-CoW (OR: 5.93, 95% CI: 2.08–16.94, *P* = 0.001) were associated with AIS after adjustment for age, sex, and clinical risk factors such as smoking, hypertension, hyperlipemia, and diabetes mellitus (Table 5).

**Table 5:** Association between vascular risk factors and acute ischemic stroke

Plaque features	Univariate regression			Multivariate regression		
	OR	95% CI	*P*	OR	95% CI	*P*
Enhancement grade (grade 1)	1.127	0.932–1.363	0.218			
Enhancement grade (grade 2)	**1.229**	**0.998–1.529**	**0.064**	**1.033**	**1.000-1.067**	**0.048**
HT_1_S (with high signal in T_1_)	1.041	0.983–1.103	0.168			
Irregularity of plaque surface (with irregularity)	**1.077**	**1.028–1.128**	**0.002**	**1.010**	**1.004–1.015**	**< 0.001**
Enhancement ratio (%)	**1.001**	**1.000–1.001**	**0.070**			
Positive remodeling (with)	**1.038**	**1.005–1.073**	**0.024**			
ARR (%)	**1.064**	**1.002–1.129**	**0.043**			
Incomplete A-CoW	1.806	0.322–2.021	0.646			
Incomplete symptom side P-CoW	**5.920**	**2.071–16.922**	**0.001**	**5.933**	**2.079–16.935**	**0.001**

*OR* odds ratio, *CI* confidence interval, *HT*_*1*_*S* high T_1_ signal, *P-CoW *posterior circle of Willis, *ARR *arterial remodeling ratio, *A-CoW *anterior circle of Willis, *P-CoW* posterior circle of Willis

## Discussion

Our study showed that the culprit plaque features varied significantly between patients with different types of CoW. Patients with incomplete A-CoW showed a higher CER, enhancement grade, and NWI for the culprit plaque features, whereas patients with incomplete symptomatic side P-CoW showed a higher probability of HT_1_S. Moreover, incomplete A-CoW was independently associated with higher enhancement grades of the culprit plaques, whereas incomplete symptomatic side P-CoW was independently associated with a higher probability of HT_1_S in the culprit plaques. Our study also demonstrated that incomplete symptomatic side P-CoW was more commonly detected in the AIS patients compared with the patients with TIA. Furthermore, our study also showed that culprit plaques with irregularity of the plaque surface, and incomplete symptomatic side P-CoW were associated with AIS. In patients with more than one intracranial plaque, the analysis was performed by including non-culprit plaques. In such cases, the results showed that all intracranial plaques with irregularity of plaque surface, enhancement grade 2, and incomplete symptomatic side P-CoW were associated with AIS.

The collateral cerebral blood flow is mainly dependent on the CoW, especially under ischemic conditions that require compensatory changes in the blood flow [38]. However, anatomical variations have been reported in the CoW among normal individuals [39]. These anatomical variations in the CoW may be genetically pre-determined and persist in all individuals since birth. The variations of CoW can alter the hemodynamics in the cerebral arteries [39, 40] and affect the wall shear stress (WSS) in the downstream artery wall [39, 41]. WSS is a widely used hemodynamic index in the study of atherosclerosis [42–44]. High WSS is associated with plaque rupture because of endothelial dysfunction and weakened plaque surface [45]. Endothelial dysfunction, neovascularization, and inflammation promote plaque enhancement because of gadolinium leakage [46]. Our study also showed that incomplete A-CoW was associated with the enhancement grade of the culprit plaques. Furthermore, our study showed that incomplete symptomatic side P-CoW was associated with HT_1_S of the culprit plaques. HT_1_S is related to intraplaque hemorrhage, which is associated with carotid plaque progression [47]. The size of intraplaque hemorrhage in the carotid artery is an independent predictor of stroke risk [48]. Although it lacked pathological proof between intraplaque hemorrhage and intracranial atherosclerotic plaques. Several studies have shown that intraplaque hemorrhage is a characteristic feature of the vulnerable intracranial atherosclerotic plaques and is associated with stroke [13, 49]. However, thrombosis can also show HT_1_S in some studies [50], which was consistent with the signal of hemorrhage. Therefore, we thought the HT_1_S in the plaque might be the hemorrhage or thrombosis or the mixture of these two components because the thrombosis can occur secondary to the hemorrhage of plaque. However, no matter the occurrence of hemorrhage or thrombosis or this mixture, it indicated that the rupture of plaque, which was always the risk factor for the stroke.

Incomplete CoW increases the risk of AIS and TIA in patients with ICAS because cerebral perfusion cannot be maintained [21, 51]. In our study, incomplete symptomatic side P-CoW was observed in 74.2% (72/97) of the study participants and was consistent with a previous report in the study of Chinese population [30]. Our study also showed that incomplete symptomatic side P-CoW was independently associated with AIS. This suggested that the posterior communicating arteries were the primary source of collateral circulation between the anterior and posterior circulation of the brain. The posterior communicating arteries are also the origin of many penetrating arteries. If patients with P-CoW variations underwent large artery stenosis or occlusion, the brain tissue was prone to be hypoxic because they lacked sufficient compensation of collateral blood flow. Chuang et al. also reported that incomplete P-CoW was a risk factor for AIS [52]. In addition, poor integrity of CoW may relate to increased recurrent stroke in patients with severe intracranial atherosclerotic stenosis [53], and also to the poor prognosis of patients with AIS [54]. For the high-risk population with incomplete P-CoW, the progress of intracranial atherosclerosis should be closely monitored to prevent ischemic stroke. For the high-risk population with incomplete P-CoW, the progress of intracranial atherosclerosis should be closely monitored to prevent ischemic stroke. The above research confirmed that intracranial atherosclerosis patients with incomplete P-CoW may benefit from early clinical intervention (including medical treatment and intravascular treatment), post-stroke treatment and management.

In our study, the culprit plaques had a higher CER and higher enhancement grade in AIS patients than those in TIA patients. The enhancement behavior of the plaque was caused by neovascularization which reflected plaque inflammation [55]. Neovascularity was associated with the accumulation of inflammatory cells (mainly macrophages and T lymphocytes), which was related to plaque vulnerability [56]. Several studies proved that the enhancement grade and enhancement ratio of culprit plaques were associated with the occurrence of stroke among patients with acute ischemic events [57, 58]. Irregular plaque surface was another vulnerable plaque feature that indicated fibrous cap rupture and was associated with symptomatic intracranial atherosclerosis [12, 59]. Our studies also demonstrated a higher risk of AIS in patients with irregular culprit plaque surface. These findings demonstrated that the structural characteristics of CoW are potential clinical predictors of AIS and TIA in patients with symptomatic ICAS. Xiao et al. found that higher NWI was associated with AIS [57]. Liang et al. demonstrated positive remodeling was closely correlated with ischemic stroke [58]. Another study focused on the culprit plaque features and hemodynamic changes in AIS and TIA populations [37]. However, their results showed intraplaque hemorrhage and the Anterograde scores were associated with patients with stroke [37]. The reasons for these different results may be explained by different enrolled patients between ours and these above studies. All the above-mentioned studies only included patients with MCA events. In addition, Xiao et al. included more than half of the patients (49, 56.3%) with 30–69% symptomatic stenosis in the MCA [57]. Furthermore, intracranial atherosclerosis often involves multiple vessel beds. *Wu et al.* found an increased number of intracranial plaques was one of the independent risk factors for recurrent stroke [16]. In our study, we evaluated all the large intracranial vessels and measured all the plaque features downstream (bilateral MCA, ACA and PCA) of CoW. We found that irregularity of plaque surfaces, enhancement grade 2, and incomplete symptomatic side P-CoW were associated with AIS, which indicated that multiple plaques may relate to multiple stenoses in the large vessels. This would reduce cerebral blood flow and increase the risk of stroke.

## Limitations

There are limitations to our study. Firstly, our study was a cross-sectional study, which needed to select a sample of subjects from a large and heterogeneous study population. Therefore, it could be susceptible to sampling bias and difficult to make a causal inference. Our study the univariable and multivariable regression analysis to control for confounding. However, other potential variables associated with the exposure and outcome might still be neglected. In our study, the clinical risk factors and the variables were chosen through works of literature and the clinical experiences of physicians. In the future, longitudinal follow-up study should be performed to evaluate the effect of the incomplete CoW on plaques and brain circulation. Secondly, our study speculated that the incomplete CoW resulted in the cerebral hemodynamic changes and induced vulnerability of the culprit plaques. However, we did not directly measure the hemodynamic changes in the brains of the study subjects and the effects of the vulnerability of the culprit plaques based on complete or incomplete CoW. Therefore, our results require further verification by computational fluid dynamics studies. Thirdly, the sample size in this study was small and may have resulted in biased results. Therefore, large sample studies are needed to confirm our results in the future.

## Conclusions

This study demonstrated that incomplete CoW, both A-CoW and P-CoW, was significantly associated with differential atherosclerotic culprit plaque characteristics. Furthermore, incomplete symptomatic side P-CoW and irregular plaque surface were independently associated with AIS.

## Supplementary Information

**Additional file 1. TableS1.** Imaging parameters of all the MRI sequences. **Fig. S1.** One case presentedwith right eye blurred vision and right limb numbness and diagnosed with TIA 3days before HR-VWI examination. **Fig. S2.** Representative multiplanar reformation (MPR) images for the vessel from the workstation. **Fig.S3.** Representativemultiplanar reformation (MPR) images showed plaques and stenosis in a 70-year-oldfemale with right lower limb weakness for 3 days. **Fig.S4.** Plaque and infundibulum signal intensity measurements. **Fig.S5.**Representative CMR images of a 47-year-old man with a history of hypertension,diabetes mellitus, and smoking who developed symptoms of paroxysmal rightupper extremity weakness and was diagnosed with TIA 2 days before the CMR. **TableS2.**Inter-reader and intra-reader reliability of the plaque characteristics andanterograde scores[wh1]  [wh1] [wh1]Response to R1-(14). **Fig. S6.** One case reported sudden slurring of speechfor 2 days before HR-WVI examination. **Fig. S7.** One case reported right upper limb weaknessfor 4 days before HR-WVI examination.

## Data Availability

The datasets used and/or analysed during the current study are available from the corresponding author on reasonable request.

## Abbreviations

A-CoW: Anterior circle of Willis
AIS: Acute ischemic stroke
ARR: Arterial remodeling ratio
CER: Contrast enhancement ratio
CoW: Circle of Willis
DSA: Digital subtraction angiography
DSC-PWI: Dynamic susceptibility contrast-enhanced perfusion weighted imaging
DWI: Diffusion weighted imaging
HR-VWI: High-resolution vessel wall imaging
HT_1_S: High T_1_ signals
ICAS: Intracranial atherosclerosis
IR-SPACE: Inversion-recovery prepared sampling perfection with application-optimized contrast using different flip angle evolutions
LA_ref_: Reference lumen area
MCA: Middle cerebral artery
MPR: Multiplanar reformation
MR: Magnetic resonance
MRA: Magnetic resonance angiography
NIHSS: National Institutes of Health Stroke Scale
NWA: Normalized wall area
NWI: Normalized wall index
P-CoW: Posterior circle of Willis
SI: Signal intensity
STAGE: Strategically acquired gradient echo
TIA: Transient ischemic attacks
TOF: Time of flight
WA_lesion_: Wall area of the lesion
WSS: Wall shear stress
