# Supplementary material for: The incomplete circle of Willis is associated with vulnerable intracranial plaque features and acute ischemic stroke
Source: J Cardiovasc Magn Reson. 2023 Apr 6;25:23. doi: 10.1186/s12968-023-00931-2 (PMC10077703; doi:10.1186/s12968-023-00931-2)
Supplement: Supplementary file 1 — Additional file 1. TableS1. Imaging parameters of all the MRI sequences. Fig. S1. One case presentedwith right eye blurred vision and right limb numbness and diagnosed with TIA 3days before HR-VWI examination. Fig. S2. Representative multiplanar reformation (MPR) images for the vessel from the workstation. Fig.S3. Representativemultiplanar reformation (MPR) images showed plaques and stenosis in a 70-year-oldfemale with right lower limb weakness for 3 days. Fig.S4. Plaque and infundibulum signal intensity measurements. Fig.S5.Representative CMR images of a 47-year-old man with a history of hypertension,diabetes mellitus, and smoking who developed symptoms of paroxysmal rightupper extremity weakness and was diagnosed with TIA 2 days before the CMR. TableS2.Inter-reader and intra-reader reliability of the plaque characteristics andanterograde scores[wh1] [wh1] [wh1]Response to R1-(14). Fig. S6. One case reported sudden slurring of speechfor 2 days before HR-WVI examination. Fig. S7. One case reported right upper limb weaknessfor 4 days before HR-WVI examination. [file 12968_2023_931_MOESM1_ESM.docx]

**Materials and Methods**

**Additional file 1: Table S1. Imaging parameters of all the MRI sequences**

| Sequences | Field of view  (mm^2^) | Repetition time  (ms) | Echo time  (ms) | Matrix size | Slice thickness  (mm) | Bandwidth  (hz/pixel) |
| --- | --- | --- | --- | --- | --- | --- |
| DWI(b=1000) | 220 × 220 | 4000 | 93 | 180 × 180 | 5.0 | 1262 |
| TOF-MRA | 250 × 204 | 21 | 3.42 | 320 × 223 | 0.9 | 185 |
| STAGE-MRA | 256 × 192 | 20 | 2.5, 12.5 | 384 × 216 | 2.0 | 650, 240 |
| DSC-PWI | 220 × 220 | 1710 | 30 | 128 ×128 | 4.0 | 521 |
| IR-SPACE | 240 × 210 | 900 | 15 | 384 × 336 | 0.55 | 465 |
| DWI= diffusion weight imaging; TOF-MRA= time of flight magnetic resonance angiography; STAGE-MRA= strategically acquired gradient echo magnetic resonance angiography; DSC-PWI= dynamic susceptibility contrast-enhanced perfusion weighted imaging; IR-SPACE= Inversion-recovery prepared sampling perfection with application-optimized contrast using different flip angle evolutions. | | | | | | |

**Evaluation of stoke mechanisms**

We categorized the stroke mechanisms after analyzing DWI, MRA, and clinical data according to previous descriptions. They included the following mechanisms: artery-to-artery embolism, local branch occlusion, in situ thrombo-occlusion, and hemodynamic impairment. The imaging appearances of these mechanisms were listed as follows:

**Artery-to-Artery Embolism**

DWI demonstrated multiple/single cortical ischemia core. It often showed scattered and was often associated with perfusion deficits throughout the territory of the stenosed vessel.

**Local Branch Occlusion**

DWI showed the ischemic core localized to an area adjacent to the stenosed vessel.

**In Situ Thrombo-occlusion**

DWI showed infarcts that extensively involve the entire or most of the stenosed arterial territory.

**Hemodynamic Impairment**

DWI showed that the ischemia core is located in border zone areas. The infarcts usually are linear in shape and are associated with perfusion deficits distal to the severely stenosed or occluded vessel.

In our study, we included 56 patients with acute ischemic stroke. Based on the above-mentioned methods, artery-to-artery embolism counted for 24/56, local branch occlusion counted for 11/56, and hemodynamic impairment counted for 21/56.

**
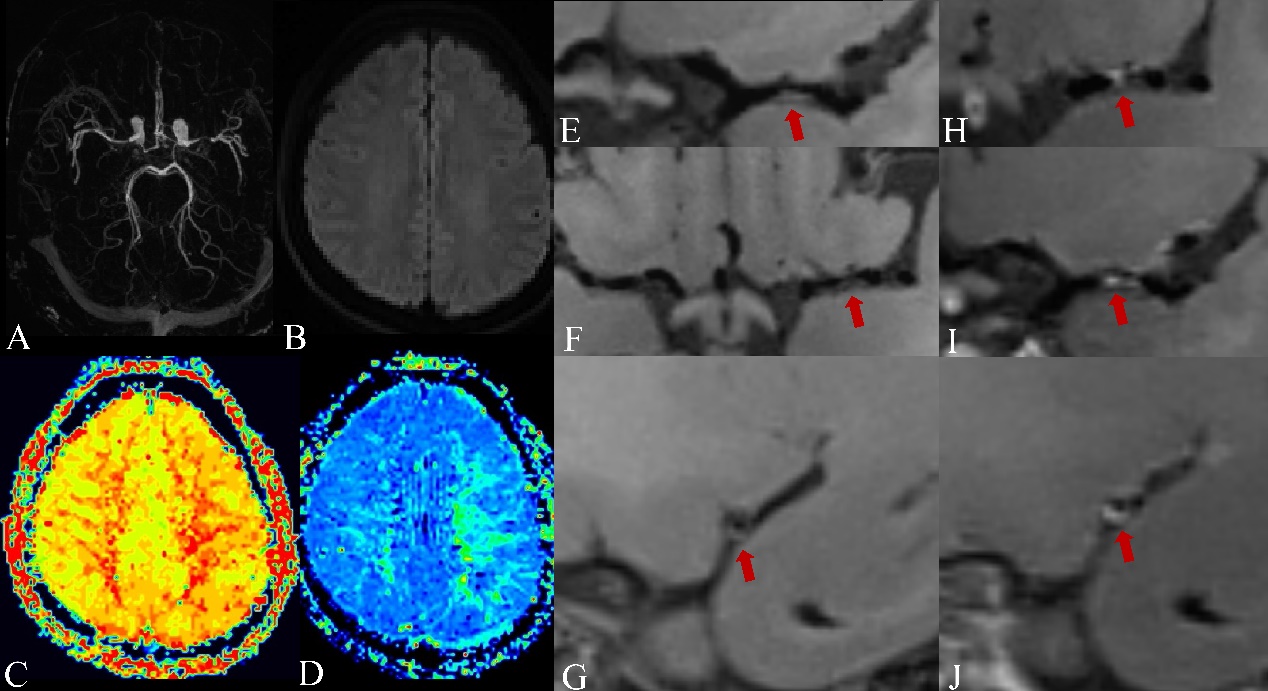
Additional file 1: Fig. S1 One case presented with right eye blurred vision and right limb numbness and diagnosed with TIA 3 days before HR-VWI examination.**

(A) showed obvious stenosis in left MCA in STAGE-MRA; (B) no positive findings presented in DWI; (C and D) TTP and MTT showed abnormal perfusion in left centrum semiovale; (E-G) pre-contrast IR-SPACE showed the culprit plaque in the left MCA; H-J the culprit plaque exhibited obviously enhanced in post-contrast IR-SPACE (red arrow).


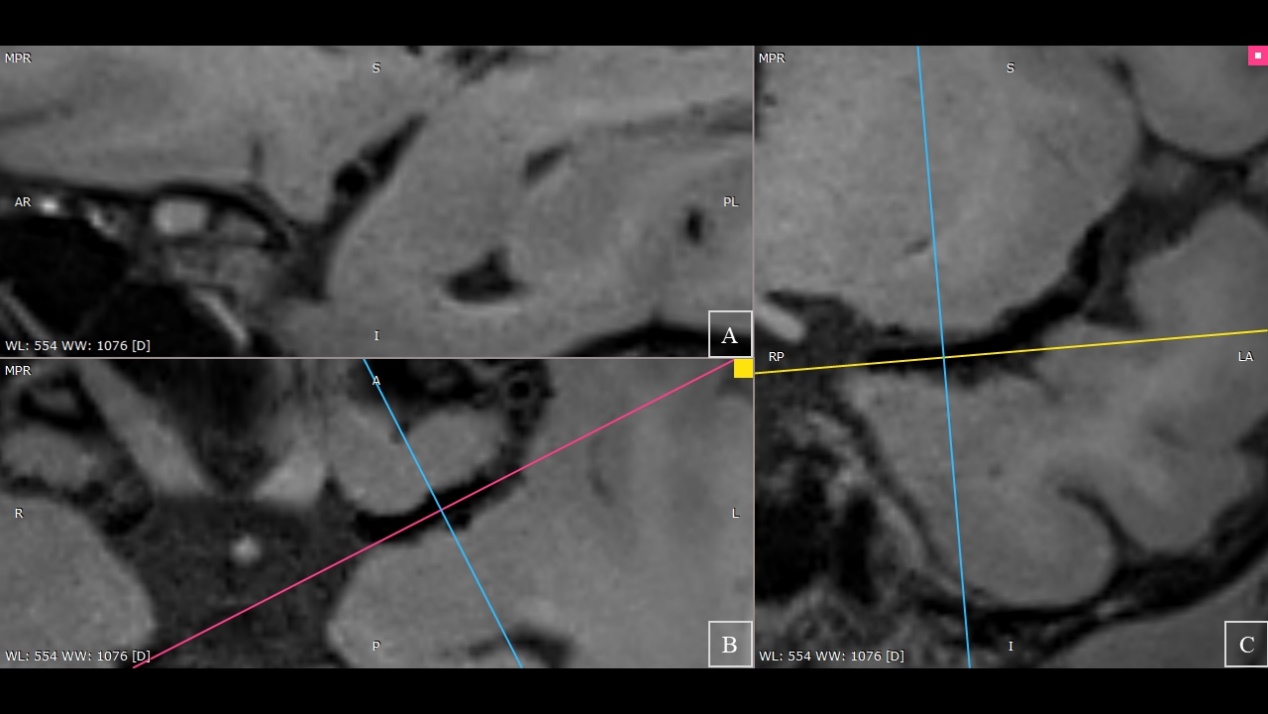


**Additional file 1: Fig. S2. Representative multiplanar reformation (MPR) images for the vessel from the workstation.**

(A) HR-VWI showed the vessel segment of the left middle cerebral artery. (B) Axial slice of a left middle cerebral artery. (C) Coronal slice of the left middle cerebral artery; All the references lines on horizontal and perpendicular positions (B and C).


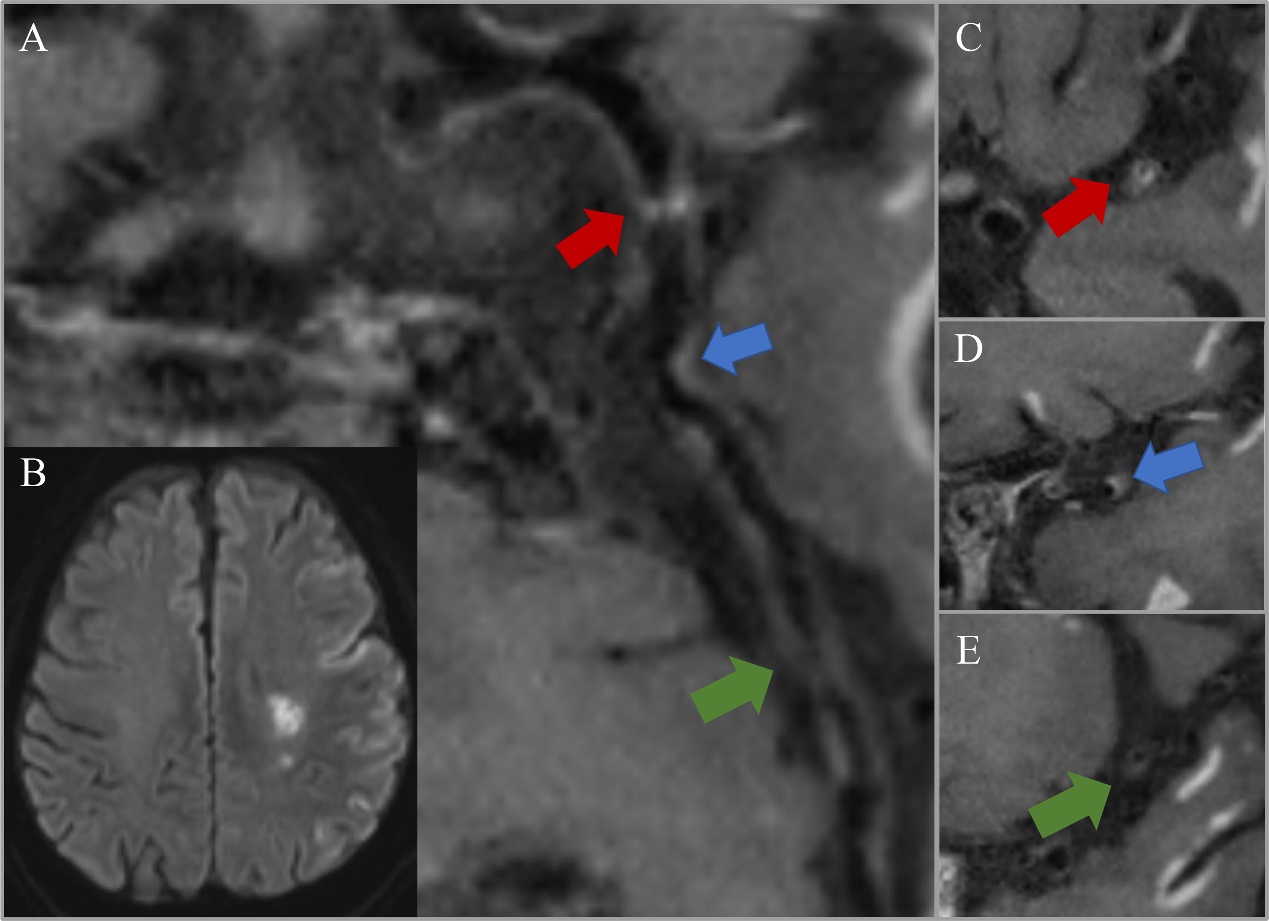


**Additional file 1: Fig. S3**. **Representative multiplanar reformation (MPR) images showed plaques and stenosis in a 70-year-old female with right lower limb weakness for 3 days.**

(A) Post-contrast HR-VWI showed multiple plaques in the left MCA. (B) DWI showed left frontal-parietal cortex and left centrum semiovale hyperintensity. (C) Culprit plaque with maximum stenosis was indicated with the red arrow. (D-E) The non-culprit plaques were indicated by the (D) blue and (E) green arrows.


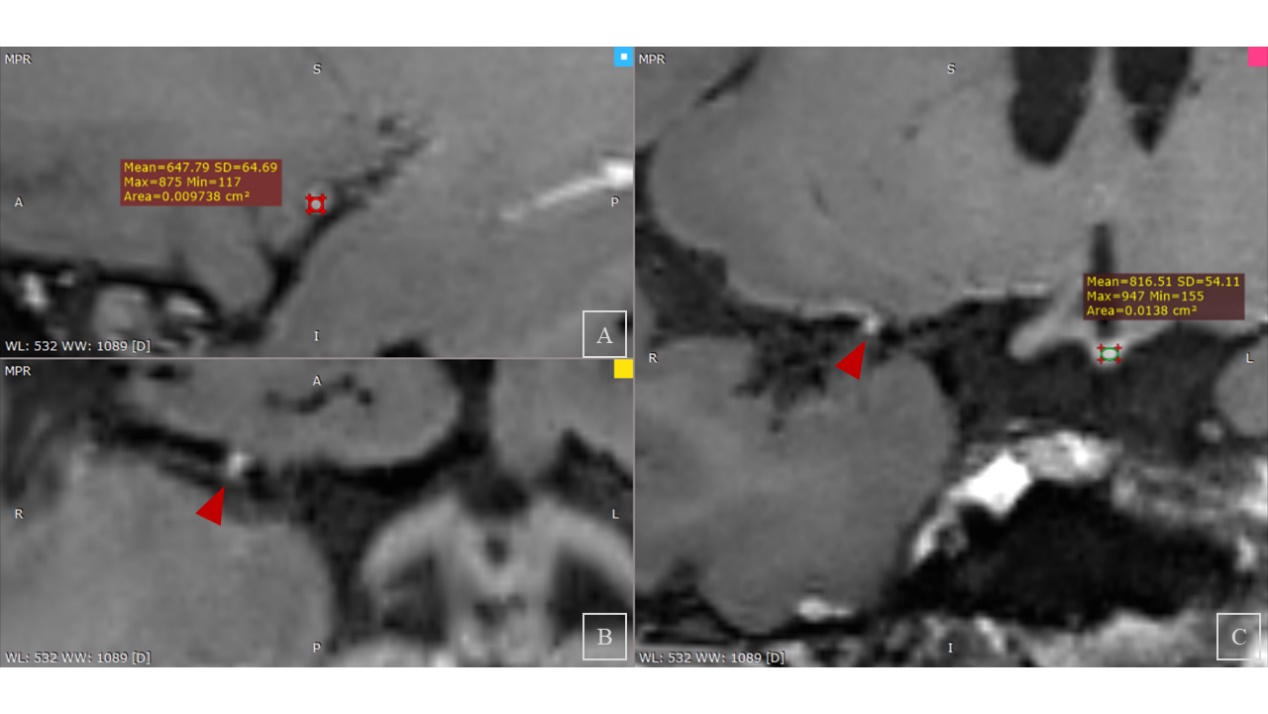


**Additional file 1: Fig. S4. Plaque and infundibulum signal intensity measurements.**

(A) The measurements of the plaque (red square) in the left MCA. (B) Axial slice of the left MCA with plaque (red arrowhead). (C) Coronal slice of the left MCA with plaque (red arrowhead) and signal intensity measurements of the infundibulum (red-green square). The plaques in the left MCA were indicated by the red arrowheads (B and C).


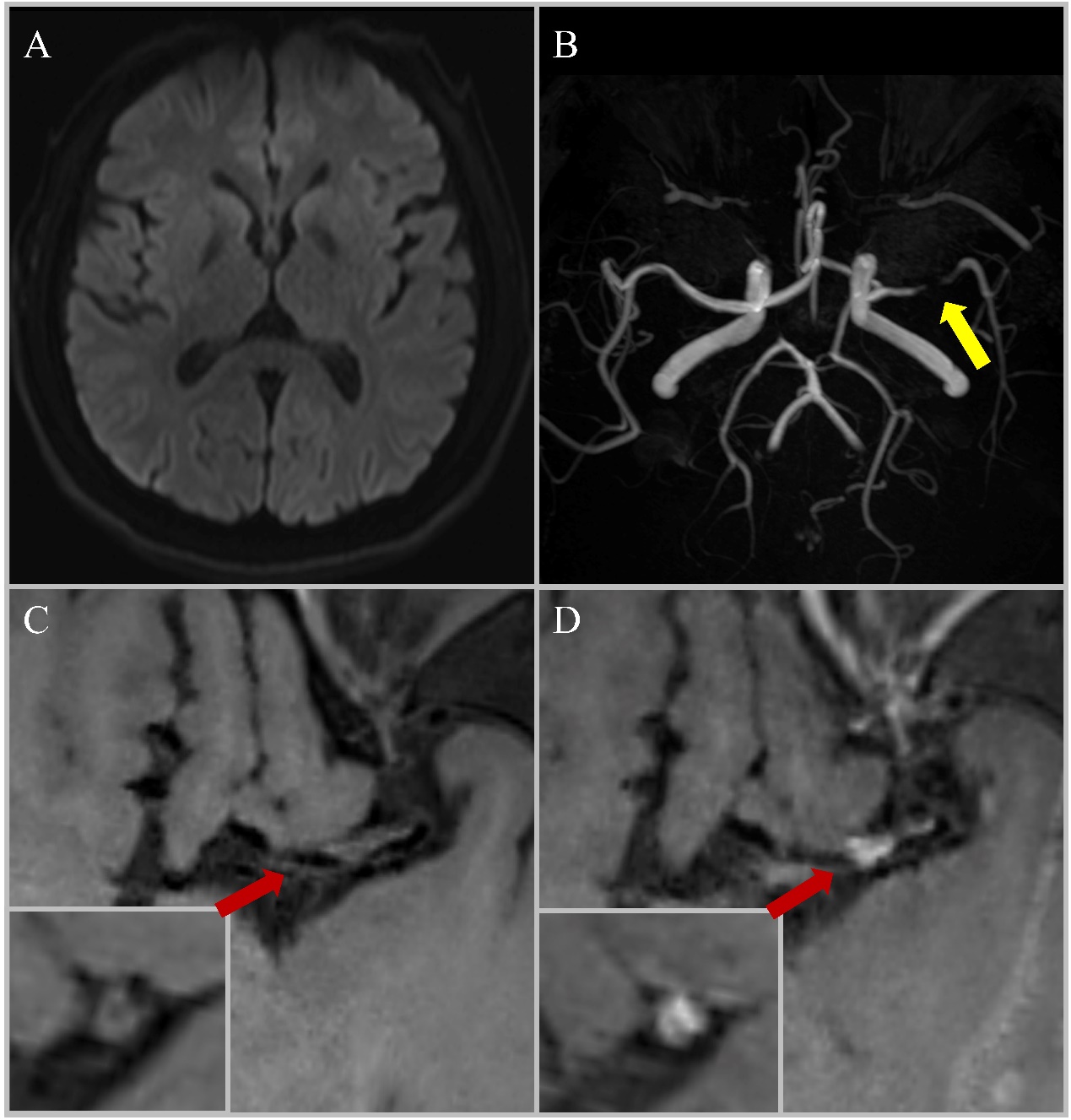


**Additional file 1: Fig. S5. Representative MRI images of a 47-year-old man with a history of hypertension, diabetes mellitus, and smoking who developed symptoms of paroxysmal right upper extremity weakness and was diagnosed with TIA 2 days before the MRI.**

(A) DWI showed no obvious signal intensity lesions. (B) TOF-MRA showed severe stenosis in the M1 segment of the left MCA. (C-D) Curved multiplanar reconstructions of the pre-(C) and post-contrast (D) HR-VWI showed a focal plaque in the left MCA with moderate stenosis (stenosis ratio=75.8%), regular plaque surface, and grade 1 enhancement (plaque enhancement ratio=1.63).

**Additional file 1: Table S2. Inter-reader and intra-reader reliability of the plaque characteristics and anterograde scores**

|  | ICC/ kappa  (Inter-reader reliability) | *P* value | ICC/ kappa  (Intra-reader reliability) | *P* value |
| --- | --- | --- | --- | --- |
| NWI | 0.991 | **< 0.001** | 0.849 | **< 0.001** |
| WA_lesion_ | 0.948 | **< 0.001** | 0.902 | **< 0.001** |
| OWA_lesion_ | 0.936 | **< 0.001** | 0.847 | **< 0.001** |
| LA_reference_ | 0.982 | **< 0.001** | 0.902 | **< 0.001** |
| ARR | 0.973 | **< 0.001** | 0.983 | **< 0.001** |
| Stenosis percentage | 0.879 | **< 0.001** | 0.727 | **0.033** |
| ER | 0.994 | **< 0.001** | 0.956 | **< 0.001** |
| A-CoW | 0.879 | **< 0.001** | 0.902 | **< 0.001** |
| P-CoW | 0.945 | **< 0.001** | 0.986 | **< 0.001** |
| HST_1_ | 0.912 | **< 0.001** | 0.936 | **< 0.001** |
| Enhanced grade | 0.936 | **< 0.001** | 0.842 | **< 0.001** |
| Positive remodeling | 0.880 | **< 0.001** | 0.862 | **0.001** |

ICC = intraclass correlation coefficient

NWI = normal wall index, ARR = arterial remodeling ratio, ER = enhancement ratio, A-CoW = anterior circle of Willis, P-CoW =posterior circle of Willis, HST_1_ = high signal in T_1_.

Reproducibility assessment was evaluated using ICC for continuous variables (NWI, WA_lesion_, OWA_lesion_, LA_reference_, ARR, stenosis percentage, ER), Weighted kappa for categorical variables (enhancement grade, positive remodeling), Cohen's kappa for binary variables (HST_1_, A-CoW, P-CoW).

**Additional file 1: Fig.**
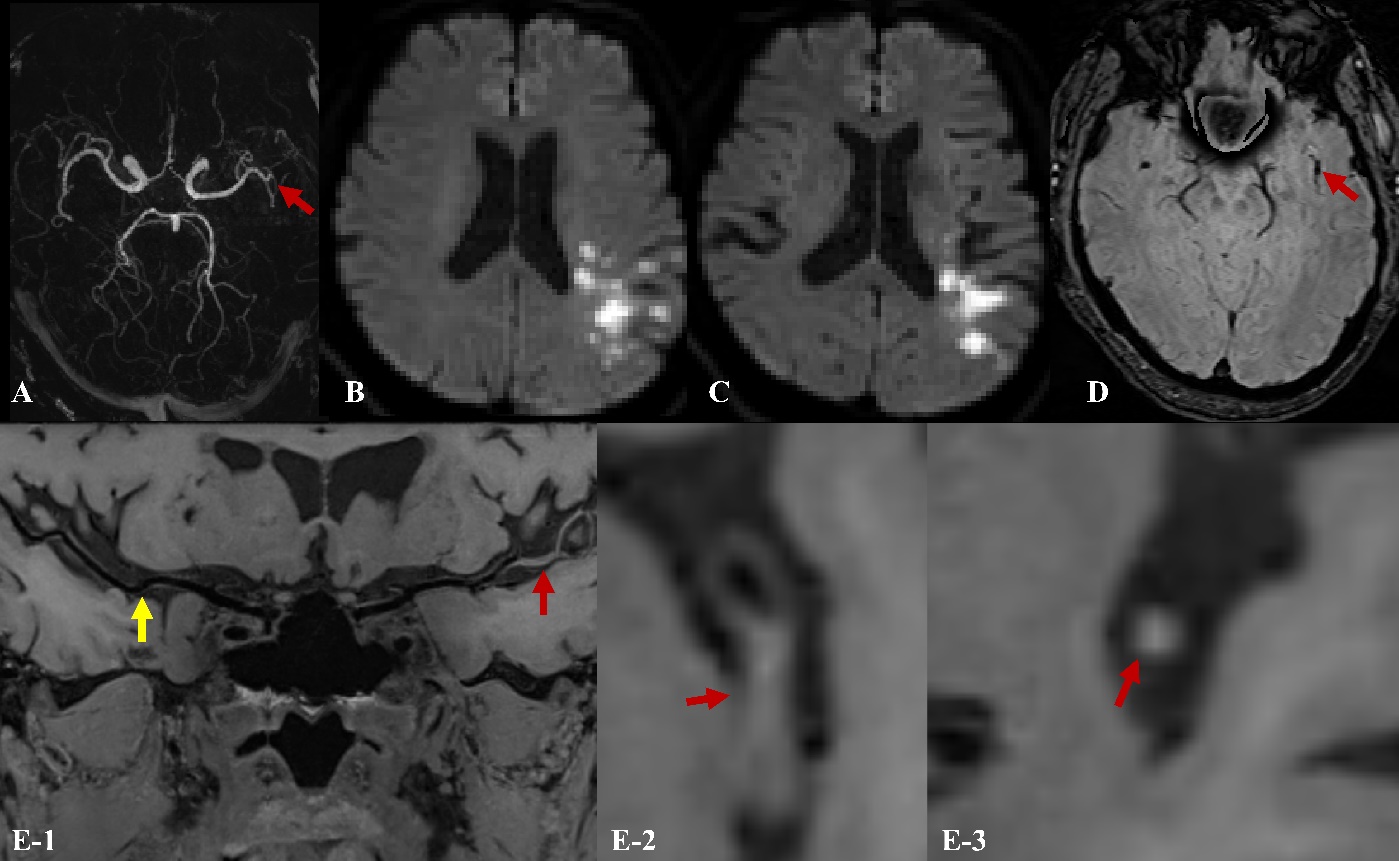
 **S6. One case reported sudden slurring of speech for 2 days before HR-WVI examination.**

(A) showed M2 segment of left MCA not displayed in STAGE-MRA (red arrow); (B-C) DWI showed patchy high signal in left parietal and insular; (D) STAGE-SWI showed strip-like low signal in the M2 segment of left MCA (red arrow); (E1-3) pre-contrast IR-SPACE showed obvious high signal in M2 segment of left MCA (red arrow) rather than the flow-void signal in right MCA (yellow arrow in E-1).

**Additional file 1: Fig.*
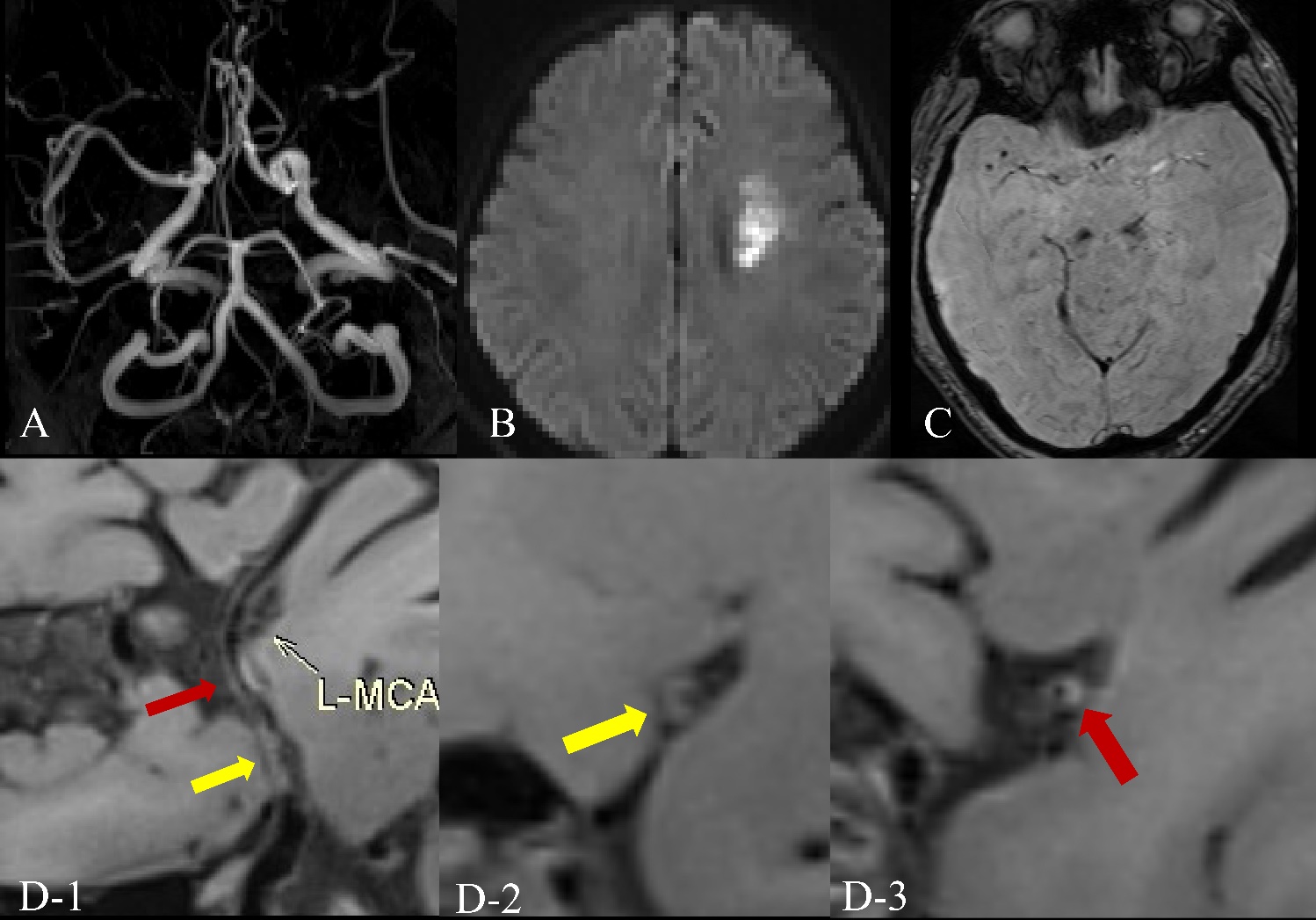
* S7. One case reported right upper limb weakness for 4 days before HR-WVI examination.**

(A) showed M1 segment of left MCA not displayed in TOF-MRA; (B) DWI showed patchy high signal in left centrum semiovale; (C) STAGE-SWI showed no abnormal signal in the M1 segment of left MCA; (D1-3) pre-contrast IR-SPACE showed two plaques in M1 segment of left MCA (red arrow and yellow arrow); (D-3) the culprit plaque showed obviously high signal in T1(red arrow).
